# Comparative bubble dynamics of plasma-mediated Nd: YAG laser-induced optical breakdown and thermally driven Er: YAG laser-activated irrigation in water

**DOI:** 10.1007/s10103-026-04999-x

**Published:** 2026-08-20

**Authors:** Yuhao Bai, Ying Zhao

**Affiliations:** Department of Dentistry, Xuan Wu Hospital of the Capital Medical University, Beijing, China

**Keywords:** Chain-like plasma, Laser-induced optical breakdown, Laser-activated irrigation, Laser-generated bubble dynamics, High-speed imaging

## Abstract

Purpose: Root-canal anatomy can limit irrigant access. Bubble dynamics in distilled water were examined for plasma-mediated 1064-nm Nd: YAG laser-induced optical breakdown (LIOB) and thermally driven 2940-nm Er: YAG laser-activated irrigation (LAI) to guide confined-model testing. Methods: LIOB was tested at 100, 200 and 300 mJ/pulse and LAI at 10, 15 and 20 mJ/pulse. Ten independent bubble events per setting were imaged at 50,000 frames/s. Projected size, lifetime, frame-averaged apparent expansion rates, aspect ratio and first rebound were compared using Kruskal–Wallis and Holm-adjusted Mann–Whitney U tests. Results: Chain-like plasma luminescence preceded LIOB bubble formation. Median primary equivalent diameters were 3.21, 4.39 and 4.72 mm in LIOB and 2.24, 2.60 and 2.89 mm in LAI. The LIOB increment was smaller at 200–300 mJ than at 100–200 mJ. Axial apparent expansion was lower and transverse expansion generally higher with LIOB. Rebound-to-primary ratios were 0.65–0.74 for LAI and 0.57–0.61 for LIOB, while absolute rebounds were larger with 200/300-mJ LIOB. Conclusion: Under the tested, non-energy-matched configurations, LIOB and LAI produced different bubble sequences and growth patterns. The results provide a free-water benchmark for confined-model experiments, but do not establish pressure safety, cleaning efficacy or superiority.

## Introduction

The aim of root canal treatment and retreatment is to reduce the intracanal microbial load and to prevent reinfection [1]. This is difficult to achieve in practice, because the root canal system contains fins, isthmuses, lateral canals and irregular cross-sections that instruments reach only in part [2, 3]. Chemical irrigation, together with the way in which the irrigant is activated, therefore remains central to the chemo-mechanical phase of disinfection [4–6].

Er: YAG laser-activated irrigation (LAI) is one established route to irrigant activation [7]. Water absorption is strong at 2940 nm, so short Er: YAG pulses can rapidly heat and vaporize liquid at or near the emitting tip; the resulting primary vapor-bubble expansion–collapse cycle drives local fluid motion, shear and pressure transients that can improve irrigant exchange relative to static syringe delivery [7–9]. The magnitude and spatial pattern of this response vary with pulse energy, pulse duration, tip design and geometric confinement [7–10].

A mechanistically different route has been explored more recently, in which the irrigant is driven not by linear water absorption but by laser-induced optical breakdown (LIOB); applied to root-canal irrigation, this approach has been described as laser-induced plasma activation (LIPA) [11, 12]. It uses tightly focused nanosecond pulses at wavelengths that water absorbs only weakly, such as 1064 nm. When the local irradiance exceeds the breakdown threshold, nonlinear ionization forms a transient plasma [13, 14]; the plasma then expands, emits a shock wave and drives cavity/bubble growth [13–15]. This differs from Er: YAG LAI, where the primary bubble originates from strong water absorption, rapid heating and vaporization at the tip [7, 10, 16]. We therefore use “laser-generated bubble” as the neutral comparative term. Where “cavitation” is retained to connect with established endodontic terminology, it is used as a descriptive umbrella term and does not imply identical nucleation mechanisms.

A commonly studied optical-breakdown configuration in water is localized focal breakdown, in which the plasma approximates a point source and the initial shock front expands radially [17]; in this geometry, acoustic energy decreases rapidly with distance [18]. Optical methods can, however, shape where energy is deposited, producing axially extended or otherwise patterned plasma instead of a strictly point-like source [19]. When the plasma source is elongated along the beam, the shock front and the ensuing bubble need not expand isotropically [20]. In a confined, canal-shaped space such directionality could in principle influence where fluid motion is generated and how it spreads from the activation site.

Laser-induced breakdown spectroscopy (LIBS; also termed laser-induced plasma spectroscopy, LIPS) provides a framework for characterizing optically generated plasmas by time-resolved emission, including estimates of electron temperature (Te) and electron number density (Ne). Solid-target studies in copper, aluminium and soil show that these quantities depend on pulse energy, wavelength, fluence and detection timing [21–26]. Although those experiments are not physically equivalent to optical breakdown in bulk water, they identify plasma variables that should be measured when interpreting a LIOB bubble response. In water, the partition of pulse energy among plasma, shock and bubble formation also depends on breakdown threshold, focal volume, optical attenuation and shielding [14, 19, 20, 27, 28]. A sub-proportional change in bubble size therefore cannot be assigned to a plateau in Te or Ne, shielding, or saturation without synchronized emission, shock and deposited-energy measurements.

The selected image descriptors were not intended as arbitrary morphological endpoints. In confined Er: YAG models, bubble oscillation timing and geometry have been examined together with intracanal pressure, bidirectional flow, irrigant exchange, debris displacement and biofilm removal [8–10, 16, 29–33]. This literature provides a rationale for measuring bubble size, lifetime, directional growth and rebound when designing downstream hydrodynamic tests. However, those relationships were established for Er: YAG activation under specific confined geometries and cannot be assumed for plasma-mediated LIOB. The unresolved question is therefore not whether one bubble appears larger in water, but whether the configuration-specific descriptors identified here predict distinct pressure, flow or cleaning responses when tested directly in the same pulp-chamber–root-canal model.

Previous work has largely characterized optical-breakdown/LIPA and erbium-laser LAI bubble behavior in separate experimental frameworks [7–12, 16, 29–34]. We therefore used a common high-speed imaging and analysis framework to compare the observable bubble sequence, projected geometry, frame-averaged dimensional expansion, lifetime and first rebound generated by 1064-nm nanosecond Nd: YAG LIOB and conventional 2940-nm Er: YAG LAI in distilled water. The wavelength, pulse duration, pulse energy, absorption, source geometry and deposited-energy fraction were not matched; accordingly, the study was designed to describe configuration-specific contrasts and to identify variables for subsequent confined-model tests, not to rank irrigation performance or isolate a mechanism effect.

## Materials and methods

### Laser systems and output characterization

LIOB was produced with a Q-switched 1064-nm Nd: YAG laser (LA-PULSAR300; MicroVec, Beijing, China) delivering 10-ns pulses at up to 15 Hz over a nominal stable range of 90–350 mJ. The arm-delivered beam had a nominal diameter of 8 mm and a nominal full-angle divergence (θ) of 2 mrad and was focused with a 50-mm-focal-length spherical lens. A nominal focal diameter of approximately 100 μm was estimated geometrically as d_est_ ≈ fθ (50 mm × 0.002 = 0.10 mm). Visible plasma luminescence was used only to locate the breakdown region, not to measure the focal diameter. The in-water beam waist was not imaged; M², refraction at the air–quartz–water interfaces, and quartz-window aberration were not characterized. The focal diameter and the corresponding nominal irradiance scale (order 10¹¹ W/cm²) are therefore theoretical order-of-magnitude descriptors rather than measured values. LAI was produced with a clinical 2940-nm Er: YAG dental laser (LightWalker, model M002-6 A/2; Fotona d.o.o., Ljubljana, Slovenia).

Output was monitored with a Vega laser power and energy meter (P/N 7Z01560; Ophir Optronics, Jerusalem, Israel) coupled to a PE50-DIF-ER-C pyroelectric energy sensor with removable diffuser (P/N 7Z02948). Pulse-train stability, including the initial pulse after activation, was verified using an oscilloscope (InfiniiVision DSOX1202A; Keysight) and matched Si (DET025A/M; Thorlabs) and InAsSb (PDA07P2; Thorlabs) photodetectors for 1064 and 2940 nm, respectively. Weak pick-off beams and wavelength-matched neutral-density filters prevented detector saturation.

### High-speed imaging and synchronization

High-speed image sequences were recorded with a FASTCAM NOVA S20 camera (Photron, Tokyo, Japan) and a 100-mm 2× macro lens (Laowa, Anhui, China), with back-illumination from a 200-W LED source (LUMINOR200A; MicroVec, Beijing, China).

For LIOB, the synchronizer (MicroPulse-825; MicroVec, Beijing, China) drove the Nd: YAG Q-switch and the camera from the same transistor–transistor-logic trigger. The sealed Er: YAG console provided no accessible emission-timing output, so the synchronizer started the camera and then requested a pulse through an electrically isolated footswitch contact closure. Thus, LIOB used direct hardware synchronization, whereas LAI used open-loop footswitch actuation and the camera was already acquiring when emission occurred. At 50,000 frames/s, consecutive images were separated by 20 µs. Device trigger latency was not used as the origin for quantitative growth or lifetime measurements; instead, image-based onset definitions were applied as described below. Nevertheless, those detection-based onsets do not reflect an identical physical nucleation event in the two mechanisms.

### Bubble imaging procedure

All experiments were carried out in a 5 × 5 × 5 cm quartz cuvette (1-mm wall thickness, JGS123 infrared-grade fused silica; ZoParon Optical, China) filled to three-quarters with laboratory-grade distilled water (Labshark; Hunan DETE Biotechnology Co., Ltd., China). For LIOB, 100, 200 and 300 mJ/pulse (15 Hz) were selected within the stable 90–350-mJ output range. Pilot observations showed repeatable elongated plasma luminescence at 90 mJ, and the three study settings sampled low, intermediate and high values within the usable range. The selected settings were specific to the observed elongated-luminescence regime and were not intended to compare its breakdown threshold with that of localized, spot-like breakdown. The beam was delivered horizontally and focused 2.5 cm from the cuvette sidewall to reduce near-wall effects. The settings were not selected to match the absorbed or deposited energy of the LAI conditions.

For LAI, irradiation was delivered through an H-14 N handpiece (Fotona d.o.o.) and a 14-mm-long, 0.4-mm-diameter flat-ended fiber tip (Flat SWEEPS 400/14; REF 84827, LOT 800742; Fotona d.o.o.). The handpiece was held above the liquid surface, and the tip was immersed 5 mm below it. The ultra-short-pulse (USP) mode (25 µs) was used at 10, 15 and 20 mJ/pulse (15 Hz), selected as lower, intermediate and upper modality-specific levels around the device’s endodontic operating parameters. They were not physically matched to the LIOB pulse energies or deposited energy. Within each modality, energy settings were acquired in a non-monotonic order; the two modalities were acquired in separate blocks because different laser and optical configurations were required.

Ten independent bubble events were analyzed per setting. This prespecified target reflected pilot event-to-event variability. For each acquisition, only the first bubble after laser activation was retained. Water temperature measured immediately before testing was 25.0 ± 0.5 °C. The distilled water was used without degassing, and dissolved-gas content was not measured. Sequences were recorded at 50,000 frames/s with 1/900,000-s exposure and stored as 12-bit grayscale TIFF files.

### Image and data analysis

Image sequences were analyzed in MicroVec software (v3.6.5; MicroVec, Beijing, China). After spatial calibration, bubble contours were segmented frame by frame with the particle-size analysis module, using predefined size ranges and detection thresholds [12]. The primary 60-event dataset was processed by a single trained engineer.

Image-analysis repeatability was assessed in a stratified random subset of 24 events (four per setting). The primary analyst repeated the analysis more than 6 months after the original assessment, and a second analyst independently processed the same events using the same SOP. The SOP, segmentation masks and software exports were archived.

Bubble dimensions were measured in a beam-referenced coordinate system, with the axial direction defined as collinear with laser propagation and the transverse direction perpendicular to it. For every frame, the full projected axial length (L_ax_), full projected transverse length (L_tr_), projected area (A) and projected-area equivalent-circle diameter (D_eq_ = 2√(A/π)) were extracted.

The representative image sequences used modality-specific time labels. For LIOB, t = 0 was the frame showing plasma luminescence, and the earliest detectable bubble contour appeared subsequently. For LAI, t = 0 was the first frame containing an identifiable vapor bubble at the fiber tip, with the preceding frame retained as baseline. For quantitative analysis in both modalities, growth time and lifetime were calculated from the first resolvable bubble contour to the maximum-size and collapse frames, respectively. This common detection rule does not make the onset physically identical across mechanisms, and onset localization was limited by the 20-µs frame interval.

The primary bubble and the first rebound bubble were analyzed separately. For each oscillation, onset, maximum size and collapse were identified; peak axial length, transverse length, projected area and equivalent diameter were recorded at maximum size, and the aspect ratio was calculated as L_ax_/L_tr_. Time to maximum and oscillation lifetime were derived from the corresponding onset, maximum-size and collapse frames. Frame-averaged apparent axial and transverse rates of full-length expansion were calculated over the image-resolved expansion interval along the beam-referenced axes and expressed in mm/ms; the equivalent-diameter expansion rate was retained as a separate, non-directional descriptor. These rates do not resolve instantaneous early acceleration or collapse. The rebound ratio was calculated within each event as the maximum projected-area equivalent-circle diameter of the first rebound bubble divided by that of the primary bubble.

### Statistical analysis

Data are presented as median [Q1–Q3]. Kruskal–Wallis H tests were followed, when significant, by two-sided Mann–Whitney U tests with within-outcome Holm correction. Omnibus effect sizes are reported as η²_H. For each of five plotted descriptors, the Holm-significant cross-modality contrast with the smallest absolute Cliff’s δ was selected for conservative reporting. Cliff’s δ = P(X_LIOB > X_LAI) − P(X_LIOB < X_LAI). Two-sided percentile-bootstrap 95% confidence intervals used 10,000 separate within-group resamples and were not adjusted for multiplicity. Positive δ denotes larger LIOB values; negative δ denotes larger LAI values. Adjusted *p* < 0.05 was considered significant. Original rank tests used IBM SPSS Statistics 19.0; revised effect-size, bootstrap and repeatability calculations used archived event-level data in Python 3.12.10 (NumPy 2.5.0; SciPy 1.18.0).

Repeatability was assessed with single-measure, absolute-agreement intraclass correlation coefficients (ICCs): a two-way mixed-effects model for intra-rater agreement [ICC(A,1)] and a two-way random-effects model for inter-rater agreement [ICC(2,1)]. Bland–Altman bias and 95% limits of agreement and absolute-error measures were interpreted alongside the ICCs. Invariant timing outcomes were summarized by exact agreement.

## Results

### Optical breakdown and plasma appearance

**Fig. 1:**
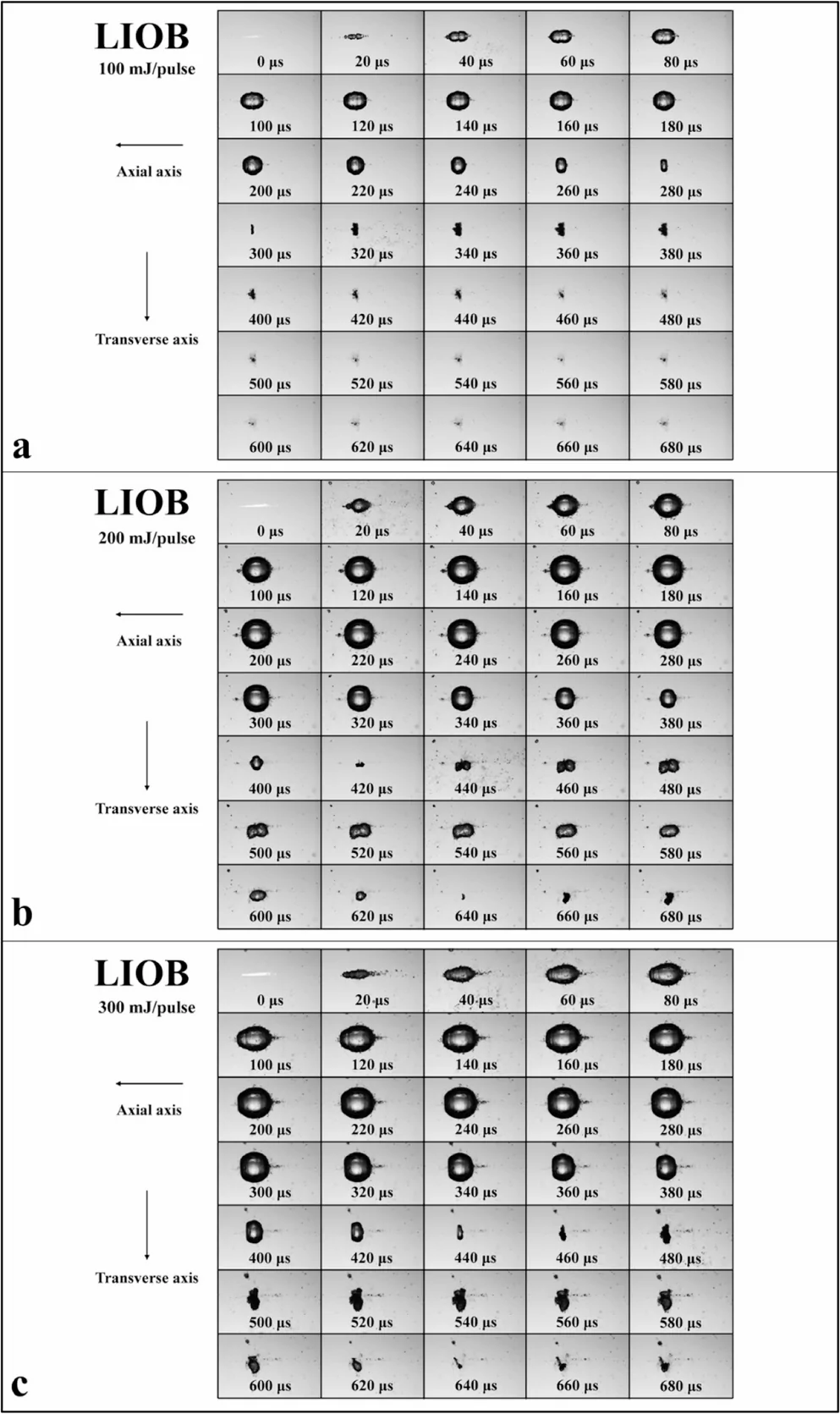
Representative high-speed image sequences of Nd: YAG laser-induced optical breakdown (LIOB) in distilled water at **a** 100 mJ/pulse, **b** 200 mJ/pulse and **c **300 mJ/pulse. Images were acquired at 50,000 frames/s with an exposure time of 1/900,000 s. t = 0 µs denotes the frame showing visible plasma luminescence; the earliest detectable bubble contour appeared subsequently. Axial and transverse directions are labeled directly in each panel, with the horizontal arrow indicating laser propagation. The luminous region appeared qualitatively more extended with pulse energy but was not quantified

**Fig. 2:**
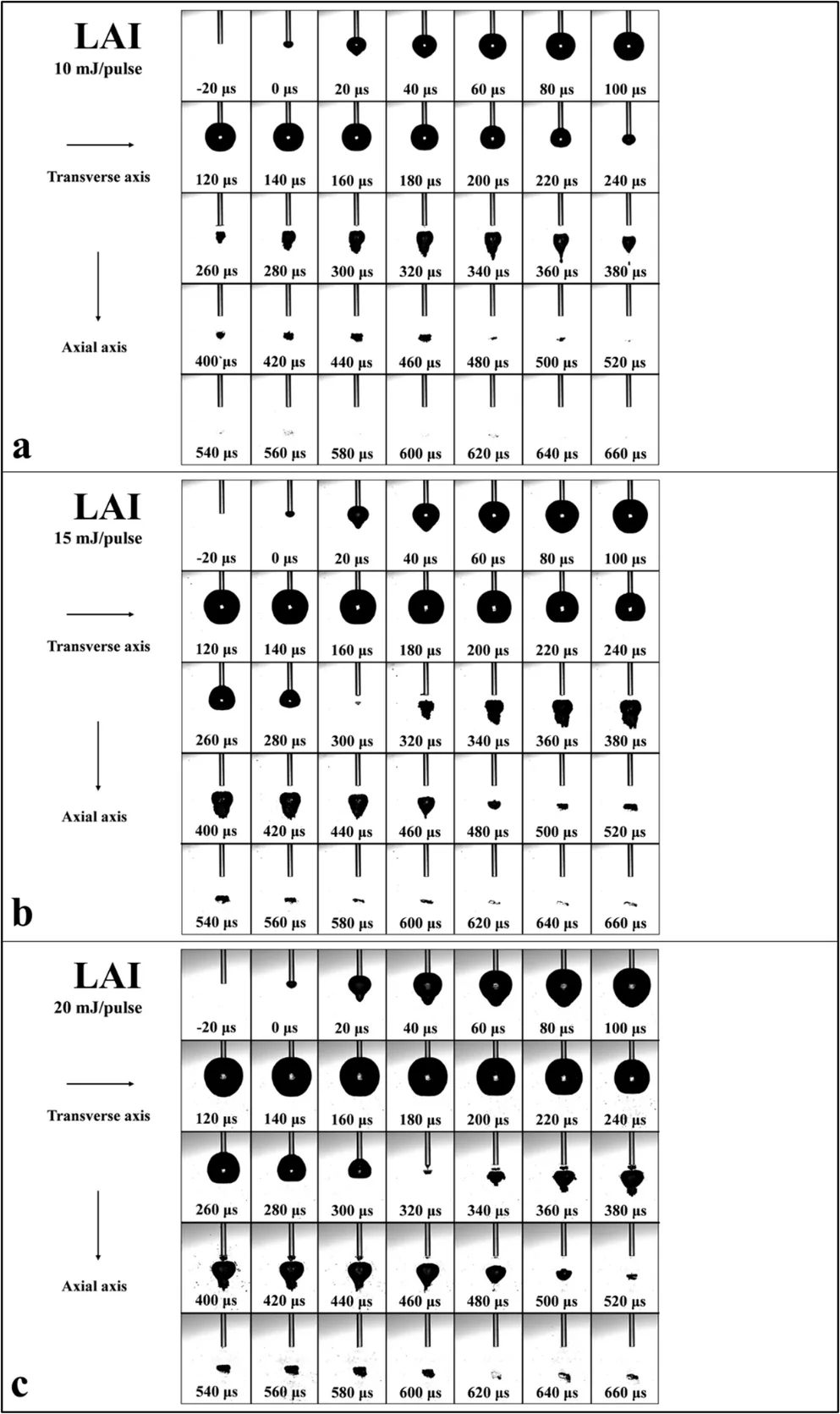
Representative high-speed image sequences of Er: YAG laser-activated irrigation (LAI) in distilled water at **a** 10 mJ/pulse, **b** 15 mJ/pulse and **c** 20 mJ/pulse, acquired at 50,000 frames/s. The frame at − 20 µs is the pre-bubble baseline and t = 0 µs denotes the first frame with an identifiable tip-associated vapor bubble. Axial and transverse directions are labeled directly in each panel; for LAI, the vertical direction is laser propagation and the horizontal direction is transverse. Primary vapor-bubble expansion, collapse and the first rebound are shown

In every LIOB group, visible chain-like plasma luminescence preceded the first resolvable bubble contour at all three pulse energies (Fig. 1a–c). In the representative sequences, the luminous region appeared qualitatively more extended as pulse energy increased from 100 to 300 mJ; its length and emission intensity were not quantified. In LAI, tip-associated vapor bubbles expanded and collapsed without visible plasma luminescence (Fig. 2a–c).

The 15 contour-derived outcomes showed high agreement: intra-rater ICCs were 0.972–0.999 and inter-rater ICCs 0.939–0.997; median absolute percentage differences were 0.61%–4.18% and 1.25%–13.00%, respectively. For primary maximum equivalent diameter, intra-rater bias was + 0.006 mm (95% limits of agreement, − 0.055 to + 0.067 mm) and inter-rater bias was − 0.005 mm (− 0.143 to + 0.133 mm); the corresponding ICCs were 0.999 (95% bootstrap CI, 0.999–1.000) and 0.997 (0.996–0.998). All four frame-derived timing outcomes showed 100% exact agreement in both comparisons.

### Primary bubble dynamics

**Fig. 3:**
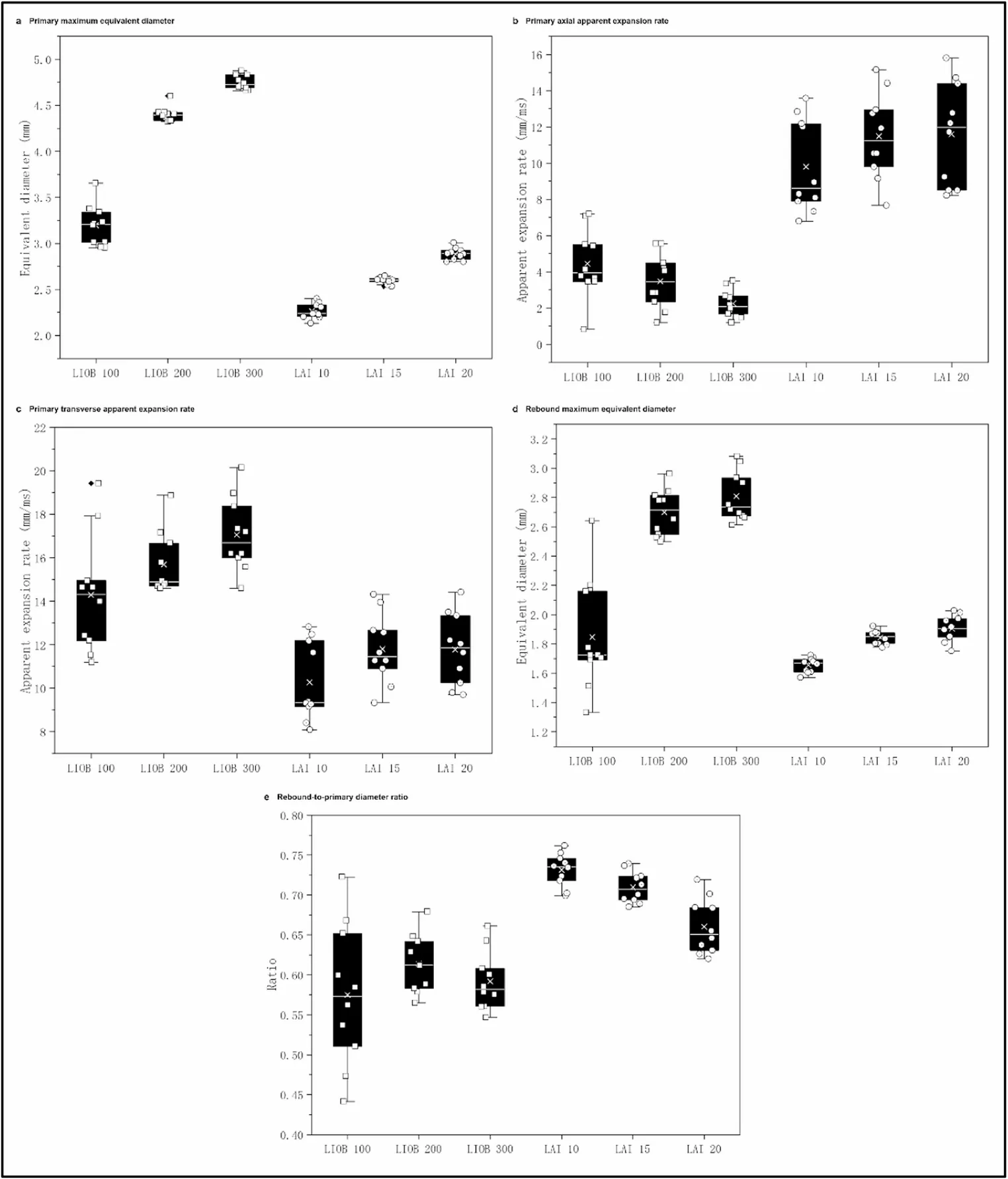
Event-level distributions of selected bubble descriptors: **a **primary maximum projected-area equivalent-circle diameter, **b **primary frame-averaged apparent axial full-length expansion rate, **c **primary frame-averaged apparent transverse full-length expansion rate, **d **first-rebound maximum equivalent diameter, and **e **rebound-to-primary maximum equivalent-diameter ratio. Boxes show the interquartile range, centre lines show medians, whiskers extend to 1.5 times the interquartile range, and × symbols show means. All 10 independent first-bubble events per setting are overlaid (open squares, LIOB; open circles, LAI). Axial and transverse directions were defined relative to laser propagation

All primary-bubble descriptors in Table 1 differed across the six tested configuration groups (all omnibus *p* < 0.001; Fig. 3). The group effect on primary maximum projected-area equivalent-circle diameter was large (H = 57.252, η²_H = 0.968, *p* < 0.001): medians increased from 3.21 to 4.39 and 4.72 mm across LIOB 100/200/300 mJ, compared with 2.24, 2.60 and 2.89 mm for LAI 10/15/20 mJ. Every pairwise comparison for this variable remained significant after within-outcome Holm correction; the smallest absolute cross-modality effect was observed for LIOB 100 mJ versus LAI 20 mJ (δ = 0.96, 95% CI 0.82–1.00). Primary growth time and lifetime also differed (H = 51.952, η²_H = 0.869, and H = 53.750, η²_H = 0.903, respectively; both *p* < 0.001). Within each modality these timing variables tended to increase with pulse energy, and LIOB at 200 and 300 mJ showed longer growth times and lifetimes than every LAI setting.

Frame-averaged apparent full-length expansion rates further distinguished the groups (Fig. 3). The axial rate was lower for LIOB (2.07–3.95 mm/ms) than for LAI (8.62–11.96 mm/ms; H = 47.023, η²_H = 0.778, *p* < 0.001), and all LIOB-versus-LAI axial-rate comparisons survived Holm correction. The smallest absolute axial-rate effect was − 0.96 (95% CI − 1.00 to − 0.82; LIOB 100 mJ versus LAI 10 mJ). The transverse rate was generally higher for LIOB (14.32–16.69 mm/ms) than for LAI (9.35–11.84 mm/ms; H = 40.387, η²_H = 0.655, *p* < 0.001); significant cross-modality contrasts mainly involved LIOB at 200 and 300 mJ, whereas LIOB at 100 mJ differed only from LAI at 10 mJ (δ = 0.76, 95% CI 0.40–0.98). At maximum expansion, LIOB bubbles were mildly elongated along the beam axis (aspect ratio 1.05–1.11), whereas LAI vapor bubbles were close to isotropic (0.94–1.00).

**Table 1:** Primary bubble dynamics generated by the LIOB and Er: YAG LAI configurations in distilled water

Metric	Unit	LIOB 100 mJ	LIOB 200 mJ	LIOB 300 mJ	LAI 10 mJ	LAI 15 mJ	LAI 20 mJ	H; η²_H	p
Maximum axial length	mm	3.43 [3.19–3.56]^c^	4.49 [4.45–4.52]^b^	4.88 [4.82–4.90]^a^	2.17 [2.14–2.26]^f^	2.55 [2.54–2.58]^e^	2.89 [2.82–2.92]^d^	57.268; 0.968	< 0.001
Maximum transverse length	mm	2.98 [2.89–3.10]^c^	4.33 [4.21–4.34]^b^	4.55 [4.54–4.67]^a^	2.31 [2.29–2.39]^e^	2.64 [2.63–2.67]^d^	2.88 [2.85–2.92]^c^	56.142; 0.947	< 0.001
Maximum projected area	mm²	8.07 [7.13–8.62]^c^	15.15 [14.81–15.33]^b^	17.52 [17.28–18.21]^a^	3.94 [3.84–4.23]^f^	5.32 [5.26–5.37]^e^	6.55 [6.31–6.69]^d^	57.252; 0.968	< 0.001
Maximum equivalent diameter	mm	3.21 [3.01–3.31]^c^	4.39 [4.34–4.42]^b^	4.72 [4.69–4.82]^a^	2.24 [2.21–2.32]^f^	2.60 [2.59–2.62]^e^	2.89 [2.84–2.92]^d^	57.252; 0.968	< 0.001
Growth time to maximum	µs	120.00 [120.00–135.00]^e^	180.00 [160.00–180.00]^b^	200.00 [185.00–200.00]^a^	120.00 [120.00–120.00]^e^	140.00 [140.00–140.00]^d^	150.00 [140.00–160.00]^c^	51.952; 0.869	< 0.001
Lifetime to collapse	µs	280.00 [260.00–280.00]^e^	380.00 [380.00–380.00]^b^	420.00 [420.00–420.00]^a^	250.00 [240.00–260.00]^f^	300.00 [285.00–300.00]^d^	320.00 [300.00–320.00]^c^	53.750; 0.903	< 0.001
Frame-averaged apparent axial expansion rate	mm/ms	3.95 [3.51–5.49]^b^	3.46 [2.47–4.42]^bc^	2.07 [1.73–2.64]^c^	8.62 [7.94–12.14]^a^	11.23 [9.98–12.89]^a^	11.96 [8.68–13.99]^a^	47.023; 0.778	< 0.001
Frame-averaged apparent transverse expansion rate	mm/ms	14.32 [12.24–14.88]^ab^	14.88 [14.71–16.45]^a^	16.69 [16.05–18.11]^a^	9.35 [9.19–12.05]^c^	11.46 [10.99–12.63]^bc^	11.84 [10.42–13.06]^bc^	40.387; 0.655	< 0.001
Aspect ratio (axial/transverse)	—	1.11 [1.05–1.17]^a^	1.05 [1.03–1.06]^a^	1.06 [1.04–1.08]^a^	0.94 [0.94–0.94]^d^	0.97 [0.96–0.97]^c^	1.00 [0.99–1.00]^b^	48.897; 0.813	< 0.001

Values are median [Q1–Q3] from 10 independent bubble events per group. H and p are from Kruskal–Wallis tests across six groups; η²_H = (H − k + 1)/(N − k). For each significant omnibus result, all 15 two-sided Mann–Whitney U comparisons were Holm-adjusted within the outcome. Groups sharing at least one superscript letter are not significantly different; groups with no shared letter differ at adjusted *p* < 0.05. Axial and transverse directions were defined relative to laser propagation.

LIOB, laser-induced optical breakdown; LAI, laser-activated irrigation; Q1–Q3, first to third quartile.

### Rebound bubble dynamics

Rebound behavior also differed among groups (Table 2; Fig. 3). Rebound maximum equivalent diameter showed a large group effect (H = 47.209, η²_H = 0.782, *p* < 0.001), with medians of 1.73, 2.72 and 2.74 mm for LIOB 100/200/300 mJ and 1.67, 1.85 and 1.91 mm for LAI 10/15/20 mJ. After Holm correction, LIOB at 200 and 300 mJ produced larger absolute rebound bubbles than every LAI setting, whereas LIOB at 100 mJ did not differ significantly from the LAI groups for this endpoint. The rebound axial apparent-rate omnibus effect was smaller (H = 13.672, η²_H = 0.161, *p* = 0.018) and did not yield any significant pairwise contrast after within-outcome correction. The rebound-to-primary diameter ratio was higher for LAI (0.65–0.74) than for LIOB (0.57–0.61; H = 41.271, η²_H = 0.672, *p* < 0.001): LAI at 10 and 15 mJ exceeded all three LIOB groups, whereas LAI at 20 mJ exceeded only LIOB at 300 mJ. The conservative representative effects were δ = 1.00 (95% CI 1.00–1.00) for absolute rebound diameter and δ = −0.80 (95% CI − 1.00 to − 0.44) for the rebound ratio. Selected cross-modality effects for all five descriptors shown in Fig. 3 are summarized in Table 3.

**Table 2:** First-rebound bubble dynamics generated by the LIOB and Er: YAG LAI configurations in distilled water

Metric	Unit	LIOB 100 mJ	LIOB 200 mJ	LIOB 300 mJ	LAI 10 mJ	LAI 15 mJ	LAI 20 mJ	H; η²_H	p
Maximum axial length	mm	1.76 [1.51–2.21]^bc^	3.10 [3.04–3.39]^a^	2.99 [2.47–3.14]^ab^	2.13 [1.98–2.19]^bc^	2.18 [2.12–2.34]^bc^	2.05 [1.99–2.25]^c^	30.768; 0.477	< 0.001
Maximum transverse length	mm	1.88 [1.47–2.07]^cd^	2.39 [2.24–2.46]^b^	2.67 [2.48–3.10]^a^	1.31 [1.27–1.34]^e^	1.53 [1.49–1.55]^d^	1.73 [1.68–1.77]^c^	51.508; 0.861	< 0.001
Maximum projected area	mm²	2.34 [2.26–3.37]^bc^	5.80 [5.14–6.18]^a^	5.87 [5.64–6.73]^a^	2.19 [2.04–2.24]^c^	2.69 [2.55–2.76]^b^	2.85 [2.68–3.05]^b^	47.209; 0.782	< 0.001
Maximum equivalent diameter	mm	1.73 [1.70–2.06]^bc^	2.72 [2.56–2.81]^a^	2.74 [2.68–2.93]^a^	1.67 [1.61–1.69]^c^	1.85 [1.80–1.88]^b^	1.91 [1.85–1.97]^b^	47.209; 0.782	< 0.001
Growth time to maximum	µs	40.00 [40.00–40.00]^b^	80.00 [65.00–80.00]^a^	80.00 [60.00–80.00]^ab^	60.00 [60.00–60.00]^b^	60.00 [60.00–80.00]^ab^	80.00 [80.00–80.00]^a^	23.677; 0.346	< 0.001
Lifetime to collapse	µs	110.00 [100.00–135.00]^c^	200.00 [185.00–200.00]^a^	200.00 [185.00–215.00]^a^	140.00 [140.00–140.00]^c^	160.00 [160.00–180.00]^b^	180.00 [180.00–180.00]^b^	45.521; 0.750	< 0.001
Frame-averaged apparent axial expansion rate	mm/ms	13.58 [12.47–15.19]^a^	13.83 [11.54–14.75]^a^	14.82 [12.51–15.56]^a^	14.66 [11.91–16.71]^a^	12.69 [11.75–13.99]^a^	9.62 [8.76–10.75]^a^	13.672; 0.161	0.018
Frame-averaged apparent transverse expansion rate	mm/ms	6.91 [4.20–7.90]^abc^	9.75 [8.40–10.12]^a^	9.10 [6.05–13.99]^ab^	5.61 [4.20–5.98]^bc^	6.39 [4.58–6.87]^bc^	4.85 [4.37–5.13]^c^	20.647; 0.290	< 0.001
Aspect ratio (axial/transverse)	—	1.12 [0.81–1.37]^bc^	1.38 [1.24–1.46]^abc^	1.11 [0.83–1.28]^c^	1.69 [1.48–1.70]^a^	1.45 [1.36–1.53]^ab^	1.19 [1.15–1.31]^c^	29.535; 0.454	< 0.001
Rebound ratio	—	0.57 [0.52–0.64]^bc^	0.61 [0.58–0.64]^bc^	0.58 [0.56–0.61]^c^	0.74 [0.72–0.74]^a^	0.71 [0.69–0.72]^a^	0.65 [0.63–0.68]^b^	41.271; 0.672	< 0.001

Values are median [Q1–Q3] from 10 independent bubble events per group. H and p are from Kruskal–Wallis tests across six groups; η²_H = (H − k + 1)/(N − k). For each significant omnibus result, all 15 two-sided Mann–Whitney U comparisons were Holm-adjusted within the outcome. Groups sharing at least one superscript letter are not significantly different; groups with no shared letter differ at adjusted *p* < 0.05. An omnibus *p* < 0.05 can occur without a significant corrected pairwise contrast. Rebound ratio is the first-rebound maximum equivalent diameter divided by the primary maximum equivalent diameter.

LIOB, laser-induced optical breakdown; LAI, laser-activated irrigation; Q1–Q3, first to third quartile.

**Table 3:** Selected pairwise effect sizes for the principal cross-modality statements

Outcome	Contrast	Cliff’s δ	95% CI	Holm p
Primary maximum equivalent diameter	LIOB 100 mJ vs. LAI 20 mJ	0.96	0.82 to 1.00	0.003
Primary apparent axial expansion rate	LIOB 100 mJ vs. LAI 10 mJ	−0.96	−1.00 to − 0.82	0.003
Primary apparent transverse expansion rate	LIOB 100 mJ vs. LAI 10 mJ	0.76	0.40 to 0.98	0.041
First-rebound maximum equivalent diameter	LIOB 200 mJ vs. LAI 20 mJ	1.00	1.00 to 1.00	0.003
Rebound-to-primary diameter ratio	LIOB 300 mJ vs. LAI 20 mJ	−0.80	−1.00 to − 0.44	0.023

Cliff’s δ is signed as Group 1 minus Group 2. Confidence intervals are two-sided percentile-bootstrap intervals based on 10,000 within-group resamples and were not adjusted for multiplicity; p values were Holm-adjusted within each outcome. For each descriptor shown in Fig. 3, the table reports the smallest absolute Cliff’s δ among Holm-significant cross-modality contrasts supporting the corresponding Results statement. The comparisons are configuration-specific and not energy-matched.

## Discussion

The image-resolved initiation sequences were consistent with different laser–water interaction pathways. In LIOB, visible plasma luminescence preceded the first resolvable bubble; in LAI, the first imageable event was a vapor bubble at the fiber tip. Nanosecond optical breakdown deposits energy into a transient plasma before shock emission and bubble growth [13–15, 17–20, 27, 28], whereas Er: YAG LAI relies primarily on strong water absorption, rapid heating and vaporization at the tip [7, 10, 29–31]. The larger LIOB bubbles observed here cannot, however, be attributed to the interaction pathway alone because pulse energy, pulse duration, absorption, focusing, source geometry and deposited energy were not matched.

The directional descriptors provide a limited view of the primary growth phase. The earliest resolvable LIOB bubbles appeared qualitatively aligned with the extended luminous source, and at maximum expansion they remained mildly elongated along the beam axis; measurable post-onset full-length expansion was nevertheless transverse-dominant. Extended plasma formation can alter the initial source geometry [18, 20]. More broadly, studies of spherical and nonspherical laser-induced bubbles show that bubble shape and oscillation depend on source geometry, boundaries and bubble interactions [35–38]. The present images do not separate these influences. Because the analysis used full projected lengths and did not track one-sided interfaces, these rates cannot be interpreted as directional volume flux or apically directed wall velocity.

The LIOB response was not proportional to nominal pulse energy over the tested range: the increase from 100 to 200 mJ was larger than the further increase from 200 to 300 mJ for several descriptors. LIBS/LIPS studies establish Te and Ne as measurable plasma descriptors and show their dependence on laser and detection parameters [21–26], but the cited solid-target plasmas do not directly establish energy partition or shielding in water. Liquid optical-breakdown studies identify focal-volume, deposited-energy and shielding effects as plausible contributors [27, 28, 39, 40]. Because plasma emission, shock amplitude and deposited energy were not quantified here, plasma shielding or saturation remains one hypothesis among changes in focal-volume breakdown, bubble deformation and unmeasured energy deposition.

Er: YAG LAI showed a different configuration-specific pattern. Its tip-associated primary vapor bubbles were smaller than the LIOB bubbles at every tested setting and shorter-lived than LIOB at 200 and 300 mJ; their maximum-expansion shapes were closer to isotropic and their axial–transverse rate balance was tighter. This is consistent with high-speed observations showing that erbium-laser LAI bubble behavior is governed by local vaporization, pulse parameters, emitter design and confinement [10, 29–32, 34]. The flat-ended tip and USP mode used here represent one tip-based LAI configuration and are not direct surrogates for PIPS or SWEEPS, which use different tip-placement and pulse-delivery strategies [7, 9, 10, 30, 31, 33]. The between-modality size difference is therefore not evidence of higher conversion efficiency or better irrigation performance.

The rebound results depended on whether absolute or relative size was considered. LAI had higher rebound-to-primary diameter ratios (0.65–0.74 versus 0.57–0.61), but LIOB at 200 and 300 mJ produced larger absolute rebounds than every LAI setting. The higher LAI ratio therefore reflected greater retention relative to the primary bubble, not a larger secondary bubble. In idealized cavitation, energy remaining at first collapse is partitioned between rebound and shock emission, while deformation can promote nonspherical collapse and jetting [35–38, 41–44]. Confined Er: YAG models also show repeated bidirectional flow [32, 33]. Neither process can be inferred from a free-water diameter ratio. The lower LIOB ratio may reflect greater first-collapse dissipation, but a two-dimensional diameter ratio is not an energy ratio and cannot identify the dissipation pathway.

Within the proposed LIPA configuration, far-field focusing aims to place optical breakdown in pulp-chamber irrigant, broadly paralleling chamber-level PIPS/SWEEPS while using a distinct plasma-mediated mechanism [7, 9–12]. A conference abstract reported preliminary PIV, pressure, temperature and smear-layer observations in separate LIPA experiments [11]; those findings do not link the present free-water descriptors to performance. Chamber-level Er: YAG studies show rapid bidirectional canal flow [32, 33]. If aligned with the tooth long axis, the extended source would be axial, whereas transverse-dominant growth suggests possible redistribution of chamber displacement and canal-orifice coupling. Yet axial expansion cannot establish lower apical pressure, and neither collapse direction nor sustained negative pressure was measured [9, 10, 29, 30]. These hypotheses require synchronized pressure and flow tests in pulp-chamber–root-canal models.

Several limitations qualify these findings. The non-energy-matched configurations preclude separating mechanism from pulse energy, duration, absorption, source position and delivery geometry. The nominal focal scale lacked M², in-water waist imaging and quantified quartz-window effects. The distilled-water cuvette did not reproduce clinical irrigants, pulp-chamber/root-canal confinement, reflected waves or anatomical resistance [8, 29–32, 34, 45]. The 20-µs interval did not resolve plasma, shock or rapid collapse. Each setting had 10 independent events without a formal a priori power calculation; repeatability used a stratified 24-event subset. Water was not degassed and dissolved gas was not measured. No deposited-energy, acoustic, pressure, flow or cleaning endpoints were obtained here. Thus efficacy, apical safety and clinical performance require confined-model testing.

## Conclusion

Under the tested, non-energy-matched conditions in water, plasma-mediated Nd: YAG LIOB and thermally driven Er: YAG LAI produced different bubble sequences. LIOB showed chain-like plasma luminescence followed by larger primary bubbles, longer primary lifetimes at 200 and 300 mJ, mild axial elongation at maximum expansion and transverse-dominant post-onset growth. Er: YAG LAI produced tip-associated, nearly isotropic primary vapor bubbles and had a higher rebound-to-primary diameter ratio; LIOB at 200 and 300 mJ produced larger absolute rebounds. These configuration-specific measurements do not establish energy-conversion efficiency, pressure safety, cleaning efficacy or clinical performance. They identify variables for direct testing in pulp-chamber–root-canal models.

## Data Availability

Data availability: The datasets generated and analyzed during the current study are available from the corresponding author on reasonable request. The high-speed imaging sequences (50,000 frames/s, 12-bit TIFF) and the bubble-dynamics measurements extracted from them are stored on institutional servers. The median values, quartiles and statistical results reported in Tables 1 and 2 are included in the manuscript.

## Abbreviations

LIOB: laser-induced optical breakdown
LAI: laser-activated irrigation
LIPA: laser-induced plasma activation
Nd:YAG: neodymium-doped yttrium aluminium garnet
Er:YAG: erbium-doped yttrium aluminium garnet
USP: ultra-short pulse
PIPS: photon-induced photoacoustic streaming
SWEEPS: shock wave enhanced emission photoacoustic streaming
TIFF: tagged image file format
LED: light-emitting diode
